# Discussing alcohol use with the GP: a qualitative study

**DOI:** 10.3399/bjgpopen20X101029

**Published:** 2020-04-29

**Authors:** Sandra Coste, Laetitia Gimenez, Aurélie Comes, Xavier Abdelnour, Julie Dupouy, Emile Escourrou

**Affiliations:** 1 General Practice Department, Toulouse III University, Toulouse, France; 2 UMR 1027 Inserm, Toulouse III University, Toulouse, France

**Keywords:** alcohol abuse, addictions, qualitative research

## Abstract

**Background:**

Despite most GPs recognising their role in the early diagnosis of alcohol use disorder (AUD), only 23% of GPs routinely screen for alcohol use. One reason GPs report for not screening is their relationship with patients; questions regarding alcohol use are considered a disturbance of a relationship built on mutual trust.

**Aim:**

To analyse the feelings and experiences of patients with AUD concerning early screening for alcohol use by GPs.

**Design & setting:**

A qualitative study of patients (*n* = 12) with AUD in remission or treatment, recruited from various medical settings.

**Method:**

Semi-structured interviews were conducted, audiorecorded, and transcribed verbatim. The authors conducted an inductive analysis based on grounded theory. The analysis was performed until theoretical data saturation was reached.

**Results:**

The participants experienced AUD as a chronic, destructive, and shameful disease. The participants expected their GPs to play a primary role in addressing AUD by kind listening, and providing information and support. If the GPs expressed a non-judgmental attitude, the participants could confide in them; this moment was identified as a key milestone in their trajectory, allowing relief and a move toward treatment. The participants thought that any consultation could be an opportunity to discuss alcohol use and noted that such discussions required a psychological and benevolent approach.

**Conclusion:**

The participants felt fear or denial from the GPs, even though they felt that discussing alcohol use is part of the GP’s job. The participants requested that GPs adopt non-judgmental attitudes and kindness when approaching the subject of alcohol use.

## How this fits in

One reason GPs report that they do not screen for AUD is their relationship with the patient, as questions about alcohol use are viewed as a disturbance in a relationship built on mutual trust. This study explored the experience of patients with AUD concerning screening for alcohol use. Patients experienced shame associated with having AUD, which pushed them to hide their disorder, and they thought screening for alcohol use was the role of GPs. If the GPs expressed a non-judgmental attitude, the patients could confide in them; this moment was identified as a key milestone in their disorder trajectory, allowing relief and movement toward treatment.

## Introduction

Worldwide, 3 million deaths occur annually due to the harmful use of alcohol, accounting for 5.3% of all deaths.^1^ Overall, 5.1% of the global burden of disease and injury, as measured in disability-adjusted life years (DALYs), is attributable to alcohol.^1^ In addition to these risks, alcohol use can lead to AUD, which is a chronic, relapsing brain disorder characterised by compulsive drug-seeking and use despite harmful consequences.^2^ France is among the countries with the highest rate of alcohol consumption worldwide.^1^ Among its 70 million inhabitants, around 5 million are daily users of alcohol, and 4.8% of 18–75 year olds display harmful patterns of alcohol use.^3^ Overall, 16% of patients who consult GPs could be experiencing an excessive use of alcohol, or AUD.^4^

In primary care populations, brief interventions and motivational interviews can reduce alcohol consumption among those with hazardous and harmful use compared with minimal or no intervention in the short and long term.^5,6^ No evidence of differences in prognosis has been shown among patients who detoxify in primary care.^7^

In most countries, GPs manage patients with substance use disorder in primary care settings.^8^ In France, patients with AUD can be managed by GPs by consulting with a specialised centre for addiction medicine as needed.^9^ In a 2008/2009 study, 52% of a sample of French GPs reported seeing patients for alcohol cessation over the prior 7 days.^10^ Three of four GPs manage their patients’ AUD, with or without consulting a specialised centre.^10^

GPs face difficulties incorporating screening and brief interventions into their routine practice.^4,11,12^ In France, only 23% of GPs routinely screen for excessive alcohol use,^10^ although most GPs recognise that the early diagnosis of AUD is part of their role.^9^ In the UK in 2009, 40% of GPs reported that they enquired about alcohol use most or all of the time.^13^ The screening and advice-giving rates seem to be approximately 30%, according to a meta-analysis of 12 trials in European and North American countries.^14^

A previous systematic review identified that the barriers to the implementation of screening in primary care reported in surveys mainly include lack of time and lack of training.^15^ Previous qualitative research has shown that whether GPs discussed issues related to alcohol use was determined by their personal relationship with alcohol and their personal qualities.^16^ In addition, one reason reported by GPs for not screening was concerns about their relationship with the patient, particularly because alcohol-related questions were considered a disturbance in a relationship built on mutual trust.^17–19^

In a recent cross-sectional survey, most adults in England agreed that healthcare providers should routinely ask about patients’ alcohol consumption.^20^ Few studies have explored the experience of the general population in qualitative ways. A recent study concerning Australian patients with or without AUD hypothesised that views of patients with AUD should differ.^21^ A better understanding of these experiences could help to ease the barriers encountered by GPs in preventing screening for AUD. To the authors’ knowledge, no qualitative study has explored the experience of patients with AUD undergoing screening for AUD.

The aim of the present study was to analyse the feelings and experiences of patients with AUD concerning early screening by GPs.

## Method

### Study design

This qualitative study adopted an inductive analysis based on grounded theory.^22^ Individual interviews seemed most appropriate for discussing AUD, which is a sensitive subject because of the stigma expressed toward it by the general population.^23^ Semi-structured interviews allowed the authors to ask open questions while offering a flexible structure.

The first draft of the interview guide was created by two researchers (AC and XA) based on the literature and the researchers’ experience. The interview guide was reviewed by two experts (JD and SC) until a consensus was reached. This guide evolved throughout the study. The first and final French interview guides are available on request. The first part of the guide collected the patients’ demographic information, and the second part addressed the following:

- perceptions of the excessive consumption of alcohol and AUD;
- feelings regarding the care pathway for AUD;
- experience with screening or diagnosis by GPs; and,
- expectations regarding early screening for AUD.

The present study was written using the COREQ reporting guideline.^24^

### Participant selection

The participants were required to have or previously have AUD, as defined by a DSM-5^25^ score ≥2, at the time of diagnosis. The participants could have been in remission or treatment. The participants were aged >18 years, volunteered to participate, and did not have another substance use disorder (expect for tobacco) or a psychotic disorder.

The authors aimed to obtain a varied sample. GPs specialising in addiction medicine, gastroenterologists, and psychiatrists practicing in areas around the city of Muret (Haute-Garonne, Midi-Pyrénées, France) were contacted either by phone or e-mail. Then, an in-person meeting was organised to explain the study and present an abridged protocol. The physicians provided the contact details of patients who were interested in participating in the study. The authors did not ask the physicians to collect the reasons for refusal from the patients who chose not to participate. If the patient agreed, the researchers called them to schedule a single appointment at a place chosen by the participant. The authors proposed to conduct the interviews at the participants’ preferred place: the participants’ home or work, a public place, the medical office of the GP, or another health structure. Then, the authors collected the reasons for refusal to participate.

At the beginning of the interview, each participant signed a written consent form. The participants were informed that they could stop the interview at any time without providing a reason. The participants were also informed of the anonymisation of the data.

An audio-recording of all interviews allowed for faithful transcription using text processing software (Microsoft Word 2016). The transcripts were not returned to the participants. Non-verbal information was transcribed. Some field notes were taken during and immediately after the interviews to record the feelings of the researcher, the context, and the participants’ attitude.

### Research team and reflexivity

Two authors, who are residents in general practice (AC and XA), conducted the interviews independently. The interviewers were supervised by two experts; one expert in qualitative studies (SC) and one expert in addiction medicine (JD). The interviewers received specific teaching on qualitative studies by the faculty.

The interviewers did not know any of the participants prior to the study’s commencement. The interviewers were introduced as researchers and not as GPs. The participants only knew that the topic of the interview was alcohol use and were blind to the aim of the study. The interviewers were both vocationally trained as GPs but attempted to overlook their profession before, during, and after the interviews. The interviewers conducted reflexivity work throughout the study.

### Analysis

From the verbatim transcripts, meaning units were identified. The meaning units were clustered into code groups that led to themes. The themes were derived from the data. Quotes, meaning units, and themes were reported on Microsoft Excel 2016. Without data from the literature, the authors could not have chosen predefined themes. The two researchers conducted a blind data analysis. Then, the researchers conferred and chose a common analysis. Each code was discussed with a supervisor (SC) for the first four interviews; subsequently, only problematic codes were discussed. After a prior thematic analysis, the authors attempted to elaborate a theory. All interviews were analysed, including those conducted using the first interview guide. The authors stopped interviews when they had reached theoretical data saturation. Theoretical data saturation was discussed by the researchers with the supervisor (SC) present.

The authors elaborated on the theory, respecting the three successive tenets of symbolic interactionism,^26^ which could be synthesised as follows: 1) humans act on things according to the meaning they attribute to them; 2) this meaning is derived or arises from the social interaction a person has with others; and 3) these senses are manipulated in, and modified through, an interpretive process used by the person to interact with the things they encounter.

## Results

### Participants

The authors invited 13 patients to participate. One participant refused to participate because of time constraints. Twelve participants were interviewed between March 2017 and September 2017. The interviews lasted a mean of 25 minutes (range 15–40 minutes). Data saturation was reached after 10 interviews and confirmed by two more interviews. The characteristics of the interviewed patients are shown in Table 1.

**Table 1.** Participant characteristics

Participant	Duration of interview, minutes	Sex	Age, years	In remission at the time of the interview	Marital status / children, *n*	Geographic origin
1	20	M	45	N	D/0	France, Haute-Garonne
2	30	M	74	Y	Ma/0	France, Haute-Garonne
3	15	M	61	Y	S/0	Spain
4	19	F	61	N	Ma/1	Algeria
5	23	F	58	N	D/0	France, Paris
6	20	M	65	N	Ma/1	France, Haute-Garonne
7	25	M	40	Y	D/0	France, Ardennes
8	23	M	38	Y	Ma/1	France, Paris
9	18	F	55	Y	Ma/3	Algeria
10	35	F	57	N	D/2	France, Mayenne
11	16	F	43	N	D/3	France, Lille
12	14	M	43	Y	D/3	France, Midi-Pyrénées

D = divorced. F = female. M = male. Ma = married. N = no. S = single. Y = yes.

### Feelings of shame

Hiding AUD is the result of the shame associated with the disease and attributed by society:

*’* *An alcoholic is less than nothing,* […] *an alcoholic is a degenerate, a point in the line.* *‘* (Participant [P]3)

Patients experienced their disease as a cause for shame. The confrontation between their self-representation and what they attribute to the representations by society of AUD reinforce this feeling:

*’Today, this disease is still a disease that is considered a shameful disease.‘* (P3)

Even with health professionals, the participants felt guilty and stigmatised; being ‘an alcoholic’ associated them with a social group viewed poorly by wider society. This taboo is also experienced because alcohol consumption is not addressed by GPs during routine consultations.

The participants felt that those around them misunderstood their illness. Feeling misunderstood and ashamed makes AUD a taboo subject that is difficult to discuss, creating loneliness. The feeling of exclusion was equally strong in relation to wider society — which, they felt, viewed them as just 'an alcoholic', without knowledge of them as a whole person — as it was in relation to their relatives. It appears that participants could have an implicit definition of themselves with a predominance of their illness.

As participants viewed and experienced their disease as shameful, they could not discuss it spontaneously. Thus, alcohol use could only be discussed following a triggering event, such as hospitalisation or anomalies on the patient’s biological balance sheet, *‘* *I started talking to him about it* [...] *when my blood test was* … *‘* (P4), and by giving limited information, *’I had talked to my attending physician but not very much, not the quantity, I said what I drank’* (P3). Step by step, the role participants gave to society and relatives to define themselves encouraged the feeling of isolation, even if not explicitly specified verbatim.

Concerning the systematic screening for alcohol use during a consultation, participants felt they might have been surprised by the subject being raised, and such a screening would not necessarily have been well accepted. Participants doubted the feasibility:

*’I don’t see an attending physician ask all his patients whether they consume alcohol in a way that is excessive’* (P1)

It is unlikely that talking about AUD in a systematic screening could destabilise the precarious balance of the relationship between the patient and the GP.

### The importance of the GP relationship

Kind listening and a trust-based relationship is expected from the GP, whose importance could be fundamental:

*’* *G* *ood initial care, good detection by the attending physicians of potential alcoholics; it is true that if, from the beginning, there could be a first intervention of the attending physician in relation to the disease, it could perhaps avoid a lot of deaths* *’* (P7)

The role of the GP was perceived as important but complex, consisting first of the early identification of people with AUD. Nevertheless, participants felt that GPs feared managing AUD. In addition, GPs’ denial of a patient’s alcohol use could limit assessments:

*’* *However, for him* [the GP]*, my consumption was not important. I was not drinking. I was not addicted.’* (P4)

One hypothesis could be that participants did not perceive the ‘goodwill role’ of GPs as beneficial to the consultation; instead they perceived a potential goodwill approach as a fear of management. The early detection of AUD by GPs was perceived as needing serious improvement. The participants highlighted the lack of training among GPs. The participants expected their GPs to be a confidant and a companion in addressing alcohol use:

*’I felt that this man, I could trust him.‘* (P5)

*’I talk about everything; it's* [the GP] *like my therapist* [...] *It's my confidant.‘* (P12)

This companionship began with attentive listening and an understanding of the patient and their illness:

*’* *I think it's really important to find a very, very good doctor who understands us* *‘* (P11)

A duty to provide information on the risks involved in the use of alcohol was mentioned:

*‘*... *she* [the GP] *has already more or less said what could happen, a cancer, a bullshit’* (P8)

The importance of the GP in accepting care could be fundamental:

*’... the role of the physician is to help him or her understand that he or she is actually sick and needs to be treated’* (P3)

When participants perceived that they needed help, they shared the truth with their GP. There was a real need to carry out a first approach, like a de-escalation of the subject:

*’*... *there I'm the one who made the move,* [...] *I didn't feel strong but* ... *I thought to myself, take a big step, that's it!‘* (P6)

Sharing information relating to AUD seemed to be difficult for the participant. Even if GPs were spontaneously cited as a potential interlocutor, the authors could use their words to recreate their perceptions of the GP’s aim: kind listener, knowledge, and first health professional that could inaugurate the care. A parallel could be made with the role of the GP as a referent. It was the health professional who started the awareness and performed the first step in care.

### The GP’s attitude and the initiation of care

With an adapted attitude, the patient could confide in the GP and begin receiving care:

*’* *If you feel his kindness, suddenly, it changes many things.* *’* (P5)

When participants encountered kind listening and lack of judgment, their defensive attitudes could be overcome. The participants could, at this time, consult with their GP for reasons specifically related to alcohol use and could take the initiative to address the subject in consultation without any restraint. If the GP discussed alcohol use in an appropriate way, there could be an opportunity to enhance patient awareness:

*’... when he made me aware of it, it seemed obvious to me.‘* (P5)

The support participants found in discussing AUD was an important step in their disease trajectory. When participants felt no shame in speaking with their families, they could feel some support. Discussing alcohol use with the GP was experienced as a relief, allowing the participants to talk about the subject more easily with others:

*’but now I can talk about it with them, with the children.‘* (P9)

Participants could then establish future objectives. When asked how GPs should address the subject of alcohol use, the participants thought that any consultation could be an opportunity. The way the subject was approached in consultation — for example, in a direct way, *‘... to take no chances. You have to be frank!‘* (P8), or indirectly, *’... not to bring it up brutally like that ... ’* (P6) — conditioned the individual's response. To begin with, knowing the patient well was felt to be an asset. Once the climate of trust is established, an attempt should be made to bring up the subject through insignificant questions. The choice of words used seemed important, while there were no specific terms to use:

*’I think he wore kid gloves because he thought it wasn't, not necessarily something you could hear easily.‘* (P5)

The participants needed to feel cared for:

*’If you feel his kindness, suddenly, it changes many things.‘* (P5)

The GP must demonstrate kind listening. The subject of alcohol must be addressed with a psychological approach. When the patient reported some alcohol consumption, hesitating to explore the subject in detail was not appropriate:

*’... ask him for his consumption, and how he consumes‘* (P3)

To ensure that the subject in consultation is approached in the best possible way, the participants strongly advised that the GP not be clumsy:

*’There are doctors, not necessarily generalists, but even addiction specialists, who are clumsily ineffective with patients.’* (P5)

It was found that there were two levels in the relationship with the GP. The first step is the establishment of an appropriate contact with their GP, opening a door that can lead to further care. Several key factors could be proposed for establishing appropriate contact: availability, trust, confidence, knowledge of the participant’s situation, and skills for AUD management. The analysis demonstrated that this first step was necessary in order to go further in establishing care and support for a patient with AUD, even if not specified by the participants.

The second step was the inauguration of a link between participants and GPs. The authors found that the link could be represented as a fine marine anchor. Once the anchorage was effective, each of the two extremities had to slowly make the effort to try to be closer in the relationship and start a care plan. The anchor could be thrown by the GP to the patient to start a link. Once the link was initiated, the GP’s attitude and skills could permit an anchorage. It appeared that if the GP was hesitant or too directive, the anchorage failed. During the care, the more the GP was able to develop a trusting environment based on their communication skills, the more the anchorage could be effective in permitting a care plan. The anchor could also be thrown by the patient who identified the GP as a potential source of help. This was possible when the GP appeared competent, available, non-judgmental, and aware of the patient’s social situation. Finally, the authors observed after analysis of the transcripts that communication skills were central for AUD care for the participants.

## Discussion

### Summary

In this qualitative study, the participants expressed shame about having AUD attributed to them by society and sometimes their family. They regretted that GPs did not always perform screening and highlighted denial and fear of discussing alcohol use among GPs. Discussing alcohol use with the GP is a crucial moment in the disease and care trajectory, and is experienced as a relief. Patients with AUD felt that any consultation should be an opportunity to discuss alcohol use. While the patients’ attitudes facing their problem differed, they answered their GP’s questions truthfully. The participants requested that GPs approach the topic in a way adapted to the patient’s personality, after establishing a climate of trust and demonstrating kind listening.

### Strengths and limitations

Each patient who volunteered was interviewed, except for one patient, who declined for reasons of availability. The authors did not seek any special patients, and selected patients who were managed in various medical settings referred by their GP. This study was interested in the role of the GP when discussing alcohol use with patients. One limitation is that it is possible that patients without a referring physician, and consequently those who were less satisfied with their GPs, would have expressed different opinions. Additionally, recruitment from medical settings did not permit the authors to include patients who were not receiving medical care, and patients engaged in medical care are unlikely to be at the same stage of their disease as patients out of care. These different stages of the disorder could be related to different perceptions of alcohol use, AUD, and relationships with health professionals. The authors regret that some interviews did not last a long time, as some patients found it difficult to talk about the subject even though the authors tried to establish a climate of trust and let the participant choose where the interview would be conducted. Additionally, the subject was intimate and patients expressed important ideas in few words, permitting the authors to establish their results

This study is novel in its investigation of patients with AUD. The authors explored participant views that could help GPs improve early screening for AUD. Recording the interviews, transcribing the interviews faithfully and rapidly after each interview, and incorporating a double-blind analysis and supervision ensured the validity of the results. The researchers engaged in reflection throughout the study to avoid projecting their representations onto the analyses.

### Comparison with existing literature

The participants' feelings of devaluation were consistent with the paradoxical attitude towards alcohol in French society. The consumption of alcohol, particularly wine, is commonplace; however, as in the present study, people with AUD may feel condemned by society, devalued, and considered offenders.^3^ Stigma against people with AUD is present in society.^23^ Asking questions about alcohol consumption is associated with a feeling of stigmatising the patient according to 16.5% of Spanish GPs.^27^ In France, alcohol remains a taboo subject.^28^

In the present study, the participants indicated that they felt fear or denial on the part of their GP. The patients reported a lack of training among GPs, which is consistent with published data.^12^ Previous studies exploring the views of patients with methadone maintenance disorders revealed that GPs need to be more proactive about alcohol screening.^29^ In 23 semi-structured interviews, Australian patients (with or without AUD) held positive views of the role of GPs in health promotion, but had reservations about engaging in discussions concerning alcohol use.^21^ Nevertheless, the participants of the present study felt that discussing alcohol use is part of the GP’s role, which is consistent with the views of GPs.^30^

The attitude of the GP is essential in permitting the patient to speak about the disease and find help. The participants suggested that GPs adopt non-judgmental attitudes and demonstrate kindness. These views were also reported by Australian participants interviewed in the study by Tam *et al*, who asked to be met with a friendly tone and a relaxed atmosphere.^21^ This attitude on the part of the GP prompts a change in the patient’s communication about the disease. When these conditions are present, the participants asked for screening at any consultation, regardless of the purpose of the visit. Indeed, systematically screening for AUD has been shown to be more effective than screening based on clinical signs in a prospective study.^31^

Finally, the present analysis revealed that communication skills were central in AUD care for the participants. A recent review^32^ synthesised studies focusing on communication skills for delivering health behaviour change conversations; only one addressed alcohol use.^33^ In this review, the authors suggested that health promotion should be considered as a special conversational task. Furthermore, alcohol screening questionnaires are reported by GPs to result in negative reactions from patients.^18^ From the present study results, the authors thus recommend patient-centred screening approaches.

### Implications for research and practice

Each consultation could be an opportunity to screen for AUD if GPs adopt non-judgmental attitudes and goodwill during discussions concerning alcohol use. GPs should be comfortable discussing alcohol use with their patients, favouring patient-centred screening approaches. Participants in the present study showed a willingness to respond honestly to their GP’s questions and believed that such discussions were appropriate given the GPs’ role in screening for AUD. Such occasions could provide great relief for patients with AUD and mark the beginning of change.
